# Determination of Boscalid and Pyraclostrobin Fungicides from Strawberries in Accordance with Green Analytical Chemistry Strategies

**DOI:** 10.3390/foods15152619

**Published:** 2026-07-27

**Authors:** Ján Hrouzek, Agneša Szarka, Dragana Šunjka, Simona Šebestová, Sanja Lazić, Svetlana Hrouzková

**Affiliations:** 1 Institute of Analytical Chemistry, Faculty of Chemical and Food Technology, Slovak University of Technology in Bratislava, Radlinského 9, 812 37 Bratislava, Slovakia; jan.hrouzek@stuba.sk (J.H.); agnesa.szarka@stuba.sk (A.S.);; 2 Department for Environmental and Plant Protection, Faculty of Agriculture, University of Novi Sad, Trg Dositeja Obradovića 8, 21000 Novi Sad, Serbia; dragana.sunjka@polj.edu.rs (D.Š.); sanja.lazic@polj.edu.rs (S.L.)

**Keywords:** boscalid, pyraclostrobin, GC-MS, QuEChERS, green analytical chemistry

## Abstract

A green variant of the mini-QuEChERS procedure was developed for the extraction of boscalid and pyraclostrobin from strawberries, these pesticides being commonly used for spraying strawberry plants. To enhance the greenness and overall environmental sustainability of the extraction method, the selection of green extraction solvent, testing of custom-made clean-up sorbents and miniaturization of the extraction procedure were studied. N-amyl acetate as a green reagent was selected as the extraction solvent. Carbonaceous sorbents made from furniture manufacture waste were prepared by pyrolyzation and proposed as a green alternative to traditionally used graphitized carbon black in the dispersive solid phase extraction clean-up step of the mini-QuEChERS procedure. The green mini-QuEChERS procedure was combined with GC-MS analysis and the method was validated. The limit of detection and quantification were 0.58 μg/kg and 1.75 μg/kg for boscalid and 4.60 μg/kg and 13.8 μg/kg for pyraclostrobin, respectively. The extraction recoveries ranged from 75% to 92%. Repeatability, expressed as the relative standard deviation was 13% and 6% for boscalid and pyraclostrobin at 25 μg/kg, 6% and 4% for boscalid and pyraclostrobin at 125 μg/kg, and 9% and 11% for boscalid and pyraclostrobin at 250 μg/kg. Within-laboratory reproducibility ranged from 4% to 12% at the three concentration levels. The analytical greenness of the method was evaluated by AGREE (0.56), Analytical EcoScale Assessment (87 points, excellent green analysis) and Complex MoGAPI (80 points). The developed and validated method was used for determination of boscalid and pyraclostrobin residues in local strawberries samples.

## 1. Introduction

Berry fruits provide many important nutrients, such as minerals, vitamins, fiber and folic acid. Their biological activity is often attributed to their polyphenolic content [1]. However, many diseases and insects attack berry fruits, which severely reduces their yield and quality, shortens their shelf-life and causes significant economic losses. Therefore, growers frequently apply pesticides to minimize the loss caused by diseases and insects. Proper and timely application of plant protection products ensures safe protection from harmful organisms, high yields, healthy crops, high-quality composition, optimal nutritional value of agricultural products and foodstuffs, labor saving in soil and crop cultivation, as well as better preservation during storage. Although pesticides are not used in large quantities (compared to mineral fertilizers or fuels), they occupy a special place as food and environmental pollutants. Pesticides represent a broad category of substances with a range of acute and chronic toxicity used in agriculture to eliminate pests, eliminate harmful fungi and control insects and weeds to sustain crop production [2,3]. The application of pesticides is inevitable owing to their contribution to boosting agricultural production. The requirement for fruits and vegetables is growing, and as a result, harvested agricultural products are being delivered to consumers heavily treated with pesticides [4]. The implemented programs of chemical plant protection are often dictated by the prevailing climatic conditions. To avoid fungal attacks, the application of fungicides, such as boscalid or pyraclostrobin, is a common practice in the field. When fresh fruit is used to prepare processed food, it will be contaminated with high probability with residues of pesticides, which may increase their concentration and affect the end product’s quality.

Boscalid is a versatile, broad-spectrum and environmentally compatible fungicide, suitable for use in high-end crops such as fruits, vegetables and horticultural plants [5]. It is effective against various pathogenic fungi, such as *Sclerotinia* spp., *Alternaria* spp. and *Botrytis* spp. [6]. Many crops, such as ornamentals, fruits and vegetables, are treated with boscalid [7]. Reports determining boscalid in cucumber [8], apples [9], soil [8], garlic [6], maize leaves and soil [10], pollen and honeybee samples [11], sweet cherries [12], grape, tomato and berry samples [13] have been reported. Pyraclostrobin has a destructive, curative and protective effect on plant pathogens both on the plant and in plant tissues [14]. Pyraclostrobin is available in various formulations. Some contain pyraclostrobin as the sole active substance (e.g., Cabrio EG; BASF Corporation, Research Triangle Park, NC). Pyraclostrobin was used against freckle disease of banana [15], banana and soil [16], citrus [17], sweet cherries [12], mango and mango juice [18], wheat [19] and rice seedlings leaves [20]. Frequently, pyraclostrobin is reported in combination with boscalid and some plant protection formulas are marketed as a premixture of these two fungicides [21], e.g., Pristine from BASF corporation [22] and Signum^®^ WG containing 26.7% boscalid + 6.7% pyraclostrobin [6]. In mixed form, fungicides have been applied for the protection of dried grape and apricot [23], in grapes and grape field soil [24], in watermelon [25] and sweet cherries [12].

However, the excessive use of pesticides affects environmental safety and causes potential threats to human health [26]. Their concentrations in berries are therefore restricted by legislation. For example, in Europe, Regulation (EC) No 396/2005 of the European Parliament and of the Council, and its subsequent modifications, have established a maximal residue limit (MRL) for boscalid of 6 mg/kg and pyraclostrobin of 1.5 mg/kg in strawberries [27]. Therefore, sensitive analytical methods are required to monitor levels of pesticide residues in food.

Sample preparation procedures are the most time-consuming part of the overall analytical process. Classical sample preparation procedures, which are time-consuming and usually performed manually, contrast with the application of fast and powerful instrumental techniques for the separation and detection of analytes. Recent advances in this field have led to innovations in existing methods and development of new techniques to save time and chemicals and improve overall performance determination. The method of extraction of semi-volatile toxic compounds, called QuEChERS extraction (acronym stands for: quick, easy, cheap, efficient, rugged, safe) represents a turning point in the field of pesticide residue analysis. For determination of boscalid and pyraclostrobin, separation and detection techniques such as high-performance liquid chromatography (HPLC) with UV or diode array detector (DAD) [6,15], HPLC with tandem mass spectrometry (MS/MS) [25], gas chromatography (GC) [28] and GC utilizing mass spectrometry (MS) detection [13] are used.

Currently, the trend in analytical chemistry is to develop new methods and modify commonly used conventional methods to meet the requirements of green analytical chemistry [29]. In these approaches, miniaturization has been a key factor in designing an integrated analytics system to ensure higher sample throughput. Using the minimum number of sample preparation steps is useful for the determination of trace and ultra-trace analytes in complex matrices, to avoid matrix effects on the determination. Design of new materials, such as multiwalled carbon nanotubes [30], nanocomposites comprising metal organic frameworks [31] and coconut fiber biochar [32] implemented in various stages of the analytical methods are contributing to increase in efficiency and robustness. Among alternative extraction solvents, n-amyl acetate represents a promising green alternative to acetonitrile owing to its favorable environmental and safety profile and suitable physicochemical properties for liquid–liquid extraction. Owing to its intermediate polarity and lipophilic character, n-amyl acetate is particularly suitable for the extraction of moderately hydrophobic pesticides such as boscalid and pyraclostrobin, while reducing the environmental burden associated with commonly used polar aprotic solvents.

Although numerous studies have investigated carbon-based sorbents, including biochar, activated carbon, carbon nanotubes and graphene, for QuEChERS clean-up, these studies have mainly focused on improving extraction efficiency using conventional extraction solvents, most commonly acetonitrile. Comparatively little attention has been paid to the simultaneous implementation of multiple green analytical strategies within a single analytical workflow, including solvent replacement, method miniaturization, the use of waste-derived sorbents and comprehensive greenness assessment. Consequently, the development of analytical procedures that combine high analytical performance with improved environmental sustainability remains an important challenge.

Increased pressure on the world’s food resources has led to heightened scrutiny and tighter regulation of potential contaminants as a means of ensuring safety and sustainability. This in turn places new demands on the analytical tools and methods used to reliably identify and quantify those contaminants. Therefore, the aim of this study was to develop a comprehensive green analytical strategy for the determination of boscalid and pyraclostrobin in strawberries. The proposed approach combines a miniaturized QuEChERS procedure, n-amyl acetate as a greener extraction solvent, and a custom-made carbonaceous sorbent prepared from recycled furniture-manufacturing waste as a sustainable alternative to conventional graphitized carbon black. In addition, instrumental conditions were optimized using GC-MS with mid-column backflushing to improve analytical throughput and reduce instrument contamination. The environmental sustainability of the complete analytical workflow was evaluated using AGREE, Analytical EcoScale and Complex MoGAPI while maintaining the analytical performance required for routine pesticide residue analysis.

## 2. Materials and Methods

### 2.1. Chemicals and Reagents

**Figure 1:**
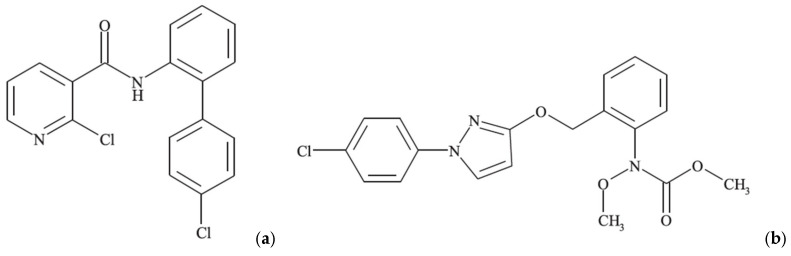
Chemical structure of fungicides under study ((**a**) boscalid, (**b**) pyraclostrobin).

Reference material of boscalid and pyraclostrobin standards was obtained from Sigma-Aldrich, Steinheim, Germany. The chemical structures of fungicides are given in Figure 1.

Pesticides were weighed into a 10 mL volumetric flask and prepared into a stock solution of individual standards in ethanol (Centralchem, Bratislava, Slovakia) at a concentration of 1 mg/mL. Composite stock solutions at a concentration of both pesticides of 20 ng/μL in acetonitrile (VWR Chemicals, Rosny-sous-Bois, France) and in n-amyl acetate (Merck KGaA, Darmstadt, Germany) were prepared. These solutions were kept in the freezer at −18 °C. The following solvents were used as extraction agents: acetonitrile (VWR Chemicals, Rosny-sous-Bois, France), ethyl acetate (VWR Chemicals, Rosny-sous-Bois, France), acetone (Chem-Lab NV, Zedelgem, Belgium), hexane (Merck KGaA, Darmstadt, Germany), ethyl-L-lactate (Thermo Fisher Scientific, Bremen, Germany), n-butyl acetate (Merck KGaA, Darmstadt, Germany), n-amyl acetate (Merck KGaA, Darmstadt, Germany), n-hexyl acetate (Thermo Fisher Scientific, Bremen, Germany), and ethanol (Centralchem., s.r.o, Bratislava, Slovakia) of pesticide grade or similar.

### 2.2. Samples

Strawberries were collected from the field in Rajka, Hungary. In total, 10 kg of strawberries were collected. Strawberries were transported to the laboratory immediately after collection, where they were decrowned, rinsed with cold water and packed into plastic food boxes, 300 g in each and packed in resealable plastic bags. After packing, strawberries were frozen and stored at −18 °C until experiment.

Before the experiment, the strawberries were defrosted and homogenized in a laboratory blender. Any homogenized strawberries that were not used were packed in a resealable plastic bag and kept frozen at −18 °C until the next experiment.

Stock strawberries did not contain any boscalid or pyraclostrobin above the limit of detection of the method and were used as a blank matrix for preparation of matrix-matched standard solutions for validation purposes. Spiked samples served for recovery determination. Blank samples were frequently analyzed in a sequence to rule out any possible contamination of the samples.

For the analysis of real samples, seven strawberry samples were purchased from local markets and major retail supermarkets in Slovakia during August 2025. The samples represented commercially available strawberries from different suppliers and were transported to the laboratory immediately after purchase. Upon arrival, the strawberries were homogenized and stored frozen until analysis.

### 2.3. Sample Preparation

Samples were prepared according to a modified mini-QuEChERS protocol. The strawberries were homogenized using a kitchen blender and 6 g of the homogenized sample was weighed into a 50 mL centrifuge tube. Subsequently, 3 mL of n-amyl acetate was added. Partitioning salts (1.2 g of MgSO_4_ and 0.3 g of NaCl) were weighed, added into the centrifuge tube, quickly capped and shaken by hand for 15 s in order to prevent MgSO_4_ clumping and to perform the extraction. Centrifugation followed at 4000 rpm for 3 min. The supernatant (2 mL) was carefully moved to a 15 mL centrifuge tube. Clean-up sorbents were weighed (300 mg of MgSO_4_, 50 mg of PSA, 50 mg of C18 and 6 mg of custom-made carbonaceous sorbent [32]) and added. The mixture was vortexed at 2500 rpm for 1 min and centrifuged at 4000 rpm for 3 min. The extract was then carefully moved into a 2 mL glass vial, 20 μL of analyte protectants (solution of 200 mg·mL^−1^ 3-ethoxy-1,2-propanediol, 20 mg·mL^−1^ D-sorbitol, 20 mg·mL^−1^ L-gulonic acid γ-lactone) [33] was added and the extract then taken for GC-MS analysis.

### 2.4. Instrumental Equipment and Conditions

Weighing of standards was carried out using the analytical balance Sartorius MC 210 P (Sartorius, Göttingen, Germany) and routine laboratory weighing was carried out with an Ohaus Navigator N-34120 balance (Nänikon Switzerland). During sample preparation, the vortex Heidolph Reax (Kelheim, Germany) and centrifuge Hettich ROTOFIX 32 A (Tuttlingen, Germany) were used. Activation and drying of sorbents were performed in a muffle furnace LMH (Hrušovany u Brna, Czech Republic) and laboratory glassware was dried in a drying oven from Binder (Tuttlingen, Germany).

Chromatographic analyses were performed using a gas chromatograph Agilent 8890 (Agilent, Little Falls, DE, USA) coupled with mass spectrometer Agilent 5977C (Agilent, Little Falls, DE, USA). The gas chromatograph was equipped with the multimode inlet (MMI) operated in solvent vent mode. Chromatographic separation was carried out using two identical columns HP-5MS UI (15 m × 0.25 mm I.D. × 0.25 μm film thickness with 5% diphenyl 95% dimethyl siloxane stationary phase) (Agilent, Little Falls, DE, USA). The carrier gas flow was set to 1.2 mL/min in column #1 and 1.4 mL/min in column # 2. Mid-column backflush was applied. During backflushing, column #1 flow was 1 mL/min in reverse flow, while column # 2 flow remained unchanged. A 1 m × 0.32 mm I. D. deactivated capillary retention gap was used. Helium (5.0, Linde Technoplyn, Bratislava, Slovakia) was used as the carrier gas. The initial inlet temperature was 120 °C held for 0.2 min. Vent pressure was 10 kPa until 0.2 min and purge flow to split vent was set to 51.75 mL/min at 1.75 min. The first temperature ramp of the inlet was 400 °C/min up to 300 °C, held for 2 min and the second temperature ramp was 400 °C/min up to 350 °C, held for 4 min. The initial oven temperature was 60 °C, held for 1.75 min. The first temperature ramp was 60 °C/min up to 150 °C, held for 0 min and the second temperature ramp was 23.8 °C/min up to 300 °C held for 5 min. The total GC run time was 14.553 min. The temperature of the transfer line to the MSD was 280 °C. The ion source of the mass spectrometer operated in electron ionization (EI) mode with ionization energy of 70 eV and temperature 230 °C. Single quadrupole was used as the mass analyzer in mass range of *m*/*z* 50–550 in scanning mode and selected *m*/*z* (140 (target ion), 112 (response percentage 29.8%), and 342 (response percentage 37.0%) for boscalid); (132 (target ion), 164 (response percentage 41.2%), and 325 (response percentage 3.9%) for pyraclostrobin) were recorded in selected ion-monitoring (SIM) mode. The qualifier response window was set to 20% for each ion. Retention time tolerance was 1% of the expected retention time. The temperature of the quadrupole was 150 °C. The ion source was cleaned with hydrogen gas using JetClean technology.

### 2.5. Method Optimization

Method optimization was performed sequentially using a one-factor-at-a-time approach. The investigated parameters included the QuEChERS procedure (Original, AOAC and CEN), extraction solvent, extraction time, shaking mode, miniaturization of the extraction procedure and particle size of the carbonaceous sorbent used during dSPE clean-up.

Extraction time was optimized by comparing manual shaking for 15, 30 and 60 s. Subsequently, the extraction mode was optimized by comparing manual shaking and vortex mixing. All optimization experiments were performed in triplicate (n = 3).

The optimum conditions were selected on the basis of extraction recovery, repeatability, ease of phase separation, the volume of extract recovered after extraction and the suitability of the extract for the subsequent dSPE clean-up and GC-MS analysis.

### 2.6. Analytical Method Validation

The analytical performance of the proposed method was evaluated according to SANTE/11312/2021 v2026 validation criteria, including trueness (apparent recovery), precision, extraction (absolute) recovery, linearity, limits of detection (LOD), limits of quantification (LOQ) and matrix effects (ME). All the experiments were performed four times.

#### 2.6.1. Sub-Sampling Variability, Extraction Recovery and Precision Evaluation

Sub-sampling variability was evaluated using five independent 6 g analytical portions taken from the same homogenized laboratory sample. Each analytical portion was separately subjected to the complete extraction and analytical procedure. The variability between the independently processed portions was expressed as the relative standard deviation under repeatability conditions (RSDr). An RSDr value not exceeding 20% was considered acceptable according to SANTE/11312/2021 v2026.

Extraction (absolute) recovery (ER) was calculated as the ratio between the peak area of analytes in samples fortified before extraction and the peak area of blank matrix extracts fortified after extraction at the same concentration (matrix-matched standards), expressed as a percentage. Mean absolute recoveries within the range of 30–140% were considered acceptable according to SANTE/11312/2021 v2026. Precision was assessed as the relative standard deviation (RSD) calculated from repeated extraction recovery experiments (n = 6) and expressed as repeatability (RSDr) and within-laboratory reproducibility (RSDwR) from spiked samples. RSDr was determined from six replicate extractions performed within a single day (n = 6), whereas RSDwR was evaluated across five consecutive days. Both accuracy and precision were determined at three concentration levels: 25 μg/kg, 125 μg/kg and 250 μg/kg.

#### 2.6.2. Linearity, Limits of Detection and Limits of Quantification Evaluation

Linearity was examined using a matrix-matched calibration approach to compensate for potential matrix-induced signal suppression or enhancement. Blank extracts were spiked at six concentration levels: 5 μg/kg, 25 μg/kg, 50 μg/kg, 125 μg/kg, 250 μg/kg, 375 μg/kg. Each calibration point represented the average of three replicate analyses of matrix-matched standards. The freshly prepared extracts were used for obtaining each point.

Calibration curves were constructed by plotting the analyte peak areas against the corresponding nominal concentrations, and linear regression was applied to evaluate the goodness of fit evaluating the coefficient of determination (R^2^). The instrumental limit of detection (LOD) and limit of quantification (LOQ) were estimated from signal-to-noise ratios of 3 and 10, respectively, using the lowest calibration standard. In accordance with the recommendations of SANTE/11312/2021 v2026, method performance (trueness and precision) was experimentally validated at fortification levels of 25, 125 and 250 μg/kg.

#### 2.6.3. Matrix Effects Calculation

Matrix effects (ME) were evaluated by comparing the slopes of matrix-matched and solvent-based calibration curves according to SANTE/11312/2021 v2026 and calculated as:

$$MF=(\frac{slope\;of\;matrix-matched\;calibration\;curve}{slope\;of\;solvent\;calibration\;curve}-1)*100\%$$

Matrix effects were categorized into the following categories:

Matrix factor in range from −20% to +20%—minimal matrix effect

Matrix factor in range from −50% to −20% or +20% to +50%—moderate matrix effect

Matrix factor under −50% or above +50%—strong matrix effect

### 2.7. Greenness Asssessment

The analytical greenness of the method was assessed using greenness evaluation metrics, namely, Analytical EcoScale Assessment [34], Complex MoGAPI [35] and AGREE [36]. For analytical greenness evaluation using Analytical EcoScale Assessment, penalty points were assigned based on the original publication written by Gałuszka et al. [34]. Complex MoGAPI was composed using the Complex MoGAPI online tool (version not provided) accessible using the link provided in the original publication [35]. AGREE assessment was generated using the AGREE software v. 0.5 2020, downloadable from the link in the original publication [36]. Input parameters for the assessment using each tool were the parameters of the final proposed method used to analyze real samples of strawberries.

### 2.8. Statistical Analysis

Statistical analysis of data was carried out using the Microsoft Excel 365 software data analysis feature. Significance level (α) was 0.05 for every statistical analysis. Number of replicates and the replicate structure are described with every result. The minimum number of replicates for any statistical analysis was n = 3. Results are expressed as mean and relative standard deviation is provided.

## 3. Results and Discussion

Strawberries represent a complex and highly perishable food matrix, which is subject to control of pesticide residue content by food control institutions throughout Europe. For this purpose, well-established procedures for isolation of pesticides, particularly QuEChERS [37], its AOAC variant [38] and the CEN variant [39] are frequently applied. In recent years, however, there has been a strong trend to reassess the approach of analytical chemists to the greenness of each individual step of the analytical method, while it is appropriate to apply new knowledge and instrumental possibilities in each area [40]. In the present work, each component of the analytical procedure was critically re-evaluated, and greener alternatives were incorporated wherever feasible.

The focus of the study was on boscalid and pyraclostrobin, commonly applied as fungicide plant protection active ingredients and sold in commercial formulations with the ratio of boscalid to pyraclostrobin at 267 g/kg:67 g/kg. To determine the optimal parameters of the method, key performance characteristics such as accuracy and precision were assessed. Suitable accuracy was achieved when the extraction recovery was in the range 70–120% and suitable precision was achieved when the relative standard deviation (RSD) was <20%, as given by SANTE/11312/2021 v2026. Method optimization was conducted using a sequential one-factor-at-a-time (OFAT) approach because the investigated parameters represented discrete methodological alternatives (e.g., QuEChERS variants, extraction solvents, sorbent particle sizes and extraction configurations) rather than continuous variables suitable for response surface optimization. Furthermore, each optimization step depended on the outcome of the preceding one, making sequential optimization the most appropriate strategy.

### 3.1. Instrumental Aspects of Separation

Instrumental parameters of the separation were investigated to increase throughput, analytical greenness and to reduce the instrument downtime caused by routine maintenance. A key modification was the implementation of mid-column backflushing, which was initiated 3 min before the end of the chromatographic run. The retention time of boscalid was 11.422 min and the retention time of pyraclostrobin was 12.140 min. Both analytes had already eluted onto column #2 three minutes before the end of the run and it was safe to reverse the flow in column 1. The application of mid-column backflush resulted in a constant baseline across analyses and prevented increase in the baseline and peak areas of all peaks, including the peaks of impurities present in the sample. Thus, backflushing effectively protected the analytical column from deposits and minimized sample carryover in subsequent injections. The optimized setup achieved a total run time of 14.55 min. In contrast, to remove impurities without backflushing, bakeout of the system using an injection of pure solvent would have more than doubled the time required for a single result up to approximately 35.48 min, including a short post-run after both runs. The use of mid-column backflushing instead of post-column backflushing reduced the time required for a single result by an additional 3.19 min, which is the duration of the backflushing phase. This means that the effective time per analysis decreased from approximately 17.74 min to 14.55 min. In total, this amounts to a 2.4× increase in sample throughput, therefore making the analysis faster and greener. In addition, the mass spectrometer was protected from the entry of impurities into the ion source. By preventing non-volatile impurities from reaching the ion source, backflushing reduces the frequency of source cleaning, thereby improving instrumental ruggedness, stability, and long-term performance.

### 3.2. QuEChERS Variants Combinations

At first, three variants of the QuEChERS method were explored to identify the procedure that provided extraction recoveries (ER) closest to 100%. The tested variants included:

Original QuEChERS—the standard procedure, using acetonitrile as the extraction solvent and a simple MgSO_4_/NaCl partitioning salt mixture [37].

AOAC Variant—an adaptation of the original QuEChERS protocol with additional buffering salts to improve extraction efficiency for pH-sensitive analytes [38].

CEN Variant—a modification recommended by the European Committee for Standardization [39,41].

As presented in Table 1, all three variants yielded comparable recoveries with no statistically significant differences. The single-factor analysis of variance (ANOVA) *p*-value for boscalid was 0.06765 and for pyraclostrobin it was 0.820337. The comparable recoveries obtained with the Original, AOAC and CEN variants indicate that the extraction of boscalid and pyraclostrobin was only weakly affected by the buffering salts. Both fungicides are chemically stable within the pH range covered by the investigated QuEChERS variants and therefore no significant differences in extraction efficiency were observed. Consequently, the simpler Original QuEChERS procedure was preferred because it provides equivalent analytical performance with lower reagent consumption and improved analytical greenness.

### 3.3. Selection and Greenness of Extraction Solvent

Several organic solvents were evaluated for their suitability in extracting boscalid and pyraclostrobin from strawberries, including acetonitrile, ethyl acetate, acetone/hexane (1:1 volume), hexane, acetone, amyl acetate and hexyl acetate. The main challenge observed with the use of these solvents was insufficient phase separation after the centrifugation. Ethyl acetate, amyl acetate and hexyl acetate produced inadequate extract volume after centrifugation for further cleanup. Centrifugation time was increased from 2 min to 5 min and later to 8 min; however, strawberries were found to form a jelly-like structure inside the test-tube and it was impossible, even with this increase in centrifugation time, to recover sufficient volume of the organic phase. The solution to ensure constant volume of solvent is further discussed in the section “Shaking during the extraction step”. Additionally, the process to recover enough extract for further use was not reproducible. Acetone extracts, in contrast, produced excessively large volumes due to incomplete separation of water from the organic phase. To remedy this issue, an additional 0.2 g of NaCl and 1 g of MgSO_4_ were added, vortexed, centrifuged and used for further clean-up. A mixture of acetone and hexane over-pressured the centrifugation test-tube after vortexing, which deformed the test-tube and rendered it inappropriate for further use. Additionally, extracts obtained with ester solvents or hexane lost their characteristic red color and instead had a slight yellow tint. Acetonitrile and acetone yielded red extracts. This color was mostly lost in the clean-up, turning to a slight pink color. Statistical evaluation using ANOVA confirmed that extraction recoveries (ERs) were solvent-dependent. However, certain solvents produced too high or too low extraction recoveries or exhibited higher variance parameters than others. Based on the results (shown in Appendix A, Table A1 and Table A2), the only solvents which provided suitable ERs for both pesticides were acetonitrile and n-amyl acetate. The poor performance of several solvents can be explained by differences in polarity and miscibility with the aqueous strawberry matrix. Ethyl acetate and higher alkyl acetates promoted the formation of stable emulsions or gel-like structures, whereas acetone exhibited excessive miscibility with water, leading to incomplete phase separation.

In addition to extraction efficiency, the environmental impact of the solvents was assessed using the Analytical EcoScale [34], which considers factors such as hazard classification and penalty points. These penalty points and hazards of the tested solvents according to the GHS are summarized in Table 2. Acetonitrile and amyl acetate not only yielded satisfactory recoveries but also scored favorably in terms of greenness. Among the tested solvents, amyl acetate was the greenest, with only 1 penalty point. Although acetonitrile remains the most frequently used extraction solvent in QuEChERS methods, n-amyl acetate represents an attractive greener alternative. Boscalid (log Kow ≈ 2.96) and pyraclostrobin (log Kow ≈ 4.0) are moderately hydrophobic fungicides, making them readily extractable into moderately non-polar organic solvents such as n-amyl acetate. Experimentally, both acetonitrile and n-amyl acetate provided satisfactory extraction recoveries, whereas the remaining solvents tested either showed poor phase separation or inadequate extraction efficiency. Since n-amyl acetate received substantially fewer penalty points in the Analytical EcoScale assessment while maintaining comparable analytical performance, it was selected as the extraction solvent for further method development.

### 3.4. Shaking During the Extraction Step

The use of a vortex to shake the centrifuge tube during the first step of the QuEChERS extraction with n-amyl acetate as the extraction solvent resulted in a highly variable and seemingly random volume of extract. Therefore, a series of experiments were performed to identify the optimal shaking method and duration. The extractable organic phase that could be comfortably withdrawn from the centrifuge tube using a pipette was weighed and its volume was calculated based on the density of n-amyl acetate. In all calculations, the laboratory temperature was 20 °C and atmospheric pressure 101.325 kPa.

First, extracts were prepared as usual. In a 50 mL centrifuge tube, 10 g of homogenized strawberries and 10 mL of n-amyl acetate were added, followed by 4 g of MgSO_4_ and 1 g of NaCl. Then, one centrifuge tube was vortexed for 30 s, and one was centrifuged for 60 s. This experiment was performed in duplicate, and the results are shown in Table 3.

A subsequent experiment was performed using the same extraction procedure as described in the previous experiment. In this case, shaking was performed by hand for 15 s. After this period, a noticeable change in the consistency of the liquid phase inside the centrifuge tube was observed; therefore, 15 s was selected as the time to be investigated (Table 4).

In further experiments, shaking was performed manually for 15 s. Extracts prepared under these conditions were collected a total of 28 times throughout the experiments, weighed, and their volumes were calculated using the density of *n*-amyl acetate (876 kg/m^3^). The average extract mass was 7.08 g, with a relative standard deviation (RSD) of 7.12% (n = 28), indicating satisfactory repeatability of the extraction procedure.

### 3.5. QuEChERS Miniaturization

To further enhance the analytical greenness of the proposed method, multiple versions of miniaturized extractions were considered and evaluated. The QuEChERS method was scaled down to use the following ratios of sample amounts and n-amyl acetate solvent volumes: 5 g/5 mL, 10 g/5 mL, 3 g/3 mL, 6 g/3 mL (n = 4). For all suggested ratios, the extraction recoveries of boscalid and pyraclostrobin were in the appropriate range (70–120%). Single-factor ANOVA of the extraction recoveries indicated no statistically significant differences among the tested miniaturization variants. Therefore, the miniaturization variant that used 6 g of sample and 3 mL of extraction solvent was selected. This variant provided an inherent pre-concentration effect while using the smallest solvent volume that still ensured reliable handling and reproducibility; smaller volumes of solvent were not investigated. In each step of the QuEChERS procedure, a small volume of solvent is lost in the solid phase due to retention in the solid phase (matrix, solid sorbents). In this investigation, it was assumed that 1 mL of solvent would be lost in every transfer of the extract. It is important to transfer clear extract and avoid pipetting of solid particles to maintain repeatability and ensure that solid particles do not enter the chromatographic system. Scaling the extraction down below 3 mL of extract would require additional sample preparation consumables, notably a vial insert, which would dramatically increase the cost per sample.

### 3.6. Carbonaceuos Sorbents

**Figure 2:**
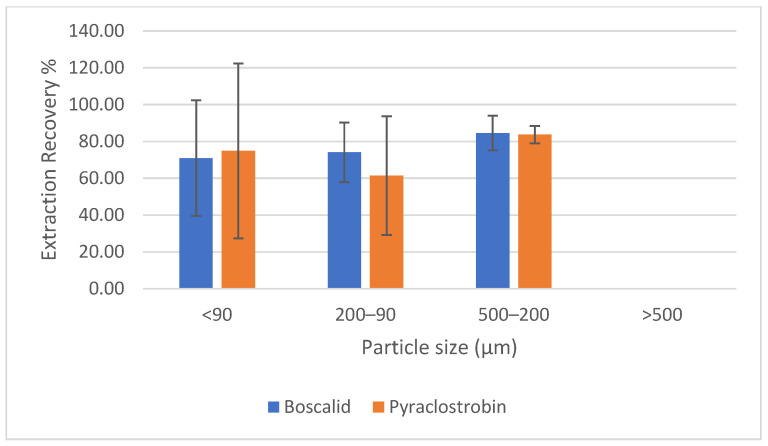
Column chart of extraction recoveries of pesticides vs. carbonaceous sorbent particle size (n = 6).

Instead of commercially available graphitized carbon black commonly used in the dispersive solid phase extraction clean-up step of the mini-QuEChERS procedure, custom-made carbonaceous sorbents were investigated. The sorbents were prepared from waste coconut fiber blocks bonded with latex, originating from mattress manufacture. The material was pyrolyzed at 500 °C in a modified muffle furnace for 120 min. After pyrolysis, the final carbonaceous material was sieved into four particle fractions: smaller than 90 μm, 200–90 μm, 500–200 μm and larger than 500 μm. All available characterization data including FTIR analysis, proximate analysis, SEM micrographs and other properties are stated in the original publication [32]. During the dSPE step of the mini-QuEChERS procedure, 3 mg/mL of the carbonaceous sorbent was used. All the particle-size fractions were evaluated in terms of extraction recovery and repeatability (Figure 2). The influence of particle size is associated with the balance between adsorption capacity and analyte retention. Fine particles provide a larger specific surface area but may retain not only matrix interferences but also the target analytes, leading to reduced recoveries. Conversely, coarse particles provide insufficient surface area for effective clean-up. The 500–200 μm fraction represented the best compromise between removal of co-extracted matrix components and preservation of the analytes in the extract. Therefore, the 500–200 μm particle-size range was selected for further experiments, as it was the only fraction that provided extraction recoveries within the acceptable range of 70–120% and RSD of ERs below 20%. Particle size >500 μm did not provide any peaks in the chromatogram and therefore the extraction recovery was considered to be zero.

### 3.7. Validation of the Method

Validation of the proposed analytical method was carried out in accordance with the requirements of the SANTE/11312/2021 v2026 guidance document [42]. The method performance was evaluated in terms of accuracy and precision, linearity, LOD, LOQ and matrix effects.

### 3.8. Selectivity

Selectivity of the proposed method was evaluated by analyzing blank strawberry matrix extracts processed according to the complete sample preparation procedure. The obtained chromatograms were examined for the presence of interfering peaks at the retention times of boscalid and pyraclostrobin. No interfering peaks or background signals that could affect analyte identification or quantification were observed, demonstrating adequate selectivity of the proposed analytical method.

### 3.9. Sub-Sampling Variability, Extraction Recovery and Precision

The effectiveness of the sample comminution and homogenization procedure was additionally assessed by evaluating sub-sampling variability. Five independent 6 g analytical portions were taken from the same homogenized strawberry laboratory sample and processed separately using the complete analytical procedure. The resulting RSDr values were 6% for boscalid and 4% for pyraclostrobin at concentration level 125 μg/kg. Both values were below the acceptance limit of 20% specified in SANTE v2026, demonstrating that the applied comminution and homogenization procedure provided sufficiently uniform and representative analytical portions. Therefore, the contribution of sub-sampling variability to the overall method variability was considered acceptable. Precision was assessed through repeatability (RSDr) and “within-laboratory reproducibility” (RSDwR) experiments at three concentration levels representative of the working range: 25 μg/kg, 125 μg/kg and 250 μg/kg. For each concentration level, five replicate analyses were performed.

The recovery studies demonstrated satisfactory extraction efficiency as summarized in Table 5. At all tested concentration levels, extraction recoveries fell within the acceptable range of 70–120%, with relative standard deviations (RSDs) below 20%. These results indicate that the proposed method fulfills the performance criteria required for pesticide residue analysis.

In addition, RSDwR was assessed over a five-day working period. The obtained recoveries and RSD values were comparable to those observed in the intra-day study, confirming the robustness and repeatability of the method over time.

### 3.10. Linearity, Limits of Detection and Limits of Quantification

The linearity of the method was studied over six concentration levels using matrix-matched calibration. The calibration curves were constructed by analyzing the fortified strawberry extracts at concentration levels 5 μg/kg, 25 μg/kg, 50 μg/kg, 125 μg/kg, 250 μg/kg and 375 μg/kg. Good linearity was achieved for both pesticides with coefficients of determination R^2^ >0.99.

The instrumental limits of detection (LOD) and quantification (LOQ) were estimated based on signal-to-noise ratios of 3 and 10, respectively. The instrumental LODs were 0.58 μg/kg for boscalid and 4.60 μg/kg for pyraclostrobin, while the corresponding instrumental LOQs were 1.75 μg/kg and 13.8 μg/kg, respectively (Table 6).

According to the recommendations of SANTE/11312/2021 v2026, the practical method LOQ should correspond to the lowest validated fortification level fulfilling the acceptance criteria for trueness and precision. In the present study, method performance was validated at fortification levels of 25, 125 and 250 μg/kg. Therefore, 25 μg/kg represents the validated practical method LOQ for both analytes.

Although the validated method LOQ is higher than the instrumental LOQ estimated from signal-to-noise ratios, it remains substantially below the current maximum residue limits (MRLs) established for strawberries (6 mg/kg for boscalid and 1.5 mg/kg for pyraclostrobin). Thus, the proposed method provides sufficient sensitivity for reliable routine monitoring of both fungicides in strawberries.

### 3.11. Matrix Effects

Matrix effects were observed as a pronounced enhancement of the analytical signal and overall method sensitivity when compared to the responses obtained from solvent-based standards. The matrix factors were calculated as the ratio of the slopes of the calibration curves in matrix and solvent. Both pesticides exhibited pronounced signal enhancement. Boscalid showed a matrix effect of +155%, whereas pyraclostrobin exhibited a matrix effect of +222%, indicating a strong enhancement of the analytical response in strawberry extracts. Matrix-induced signal enhancement is a well-recognized phenomenon in GC-MS analysis of complex food matrices. It is generally attributed to the presence of co-extracted matrix constituents, such as sugars, organic acids, pigments and other naturally occurring compounds, which compete with analytes for active adsorption sites in the injector liner, chromatographic column and transfer line. As a consequence, analyte adsorption and thermal degradation at these active sites are reduced, resulting in increased analyte transfer into the chromatographic system and enhanced detector response. This effect is particularly pronounced in fruit matrices because of their complex chemical composition.

The dispersive solid-phase extraction clean-up based on PSA, C18 and the custom-made carbonaceous sorbent considerably improved extract cleanliness by removing a substantial proportion of co-extracted matrix constituents. PSA primarily removes organic acids, sugars and other polar compounds, whereas C18 effectively retains lipophilic interferences. The waste-derived carbonaceous sorbent further contributes to the removal of pigments and other high-molecular-weight hydrophobic matrix constituents owing to its porous aromatic carbon structure and high specific adsorption capacity. In general, carbonaceous sorbents remove matrix interferences through a combination of hydrophobic interactions, π–π interactions between aromatic carbon domains and planar organic molecules, and physical adsorption within the porous structure. These mechanisms preferentially retain pigments, waxes and other high-molecular-weight co-extractives that would otherwise contribute to matrix-induced signal enhancement [43]. Nevertheless, complete elimination of matrix components was not achieved, as demonstrated by the pronounced residual matrix effects observed for both fungicides.

The stronger matrix effect observed for pyraclostrobin compared with boscalid is likely related to differences in their physicochemical properties and their interactions with residual matrix constituents during the injection process. Although the clean-up procedure substantially reduced matrix interferences, the remaining co-extractives still influenced the analytical response. Therefore, matrix-matched calibration was essential to compensate for matrix-induced signal enhancement and to ensure accurate quantification of both fungicides.

### 3.12. Analytical Greenness

**Figure 3:**
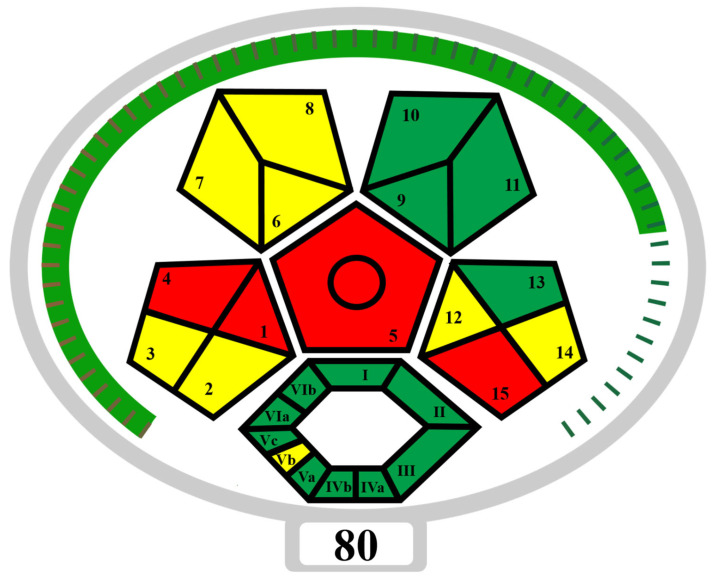
Complex MoGAPI greenness assessment of the proposed method (see details in Section 3.12 and ref. [35]).

The development of environmentally friendly analytical methods has become increasingly important in modern analytical chemistry. Therefore, the analytical greenness of this method has been assessed using various approaches, including Complex MoGAPI [35], AGREE [36] and Analytical Eco-Scale [34]. An overview of analytical methods for the analysis of boscalid and pyraclostrobin together with their greenness evaluation is provided in Table 7. The proposed method was evaluated using Complex MoGAPI, AGREE and Analytical EcoScale Assessment. This method intends to improve on its analytical greenness rating by using n-amyl acetate as a greener extraction solvent and miniaturization of the process, therefore saving reagents and sample. Comparison of this method to the original QuEChERS is provided in Table 7. As stated in Table 7, the proposed method is greener according to the AGREE score with a score of 0.56, while other analytical procedures’ scores are in the range of 0.37 to a maximum of 0.45. Evaluation of the proposed method using Complex MoGAPI gives a score of 80 (Figure 3). Points were lost in transport and storage, as well as being an indirect method requiring extraction. Points were gained in the scale of the method, using <10 mL of solvent and <10 g of sample. Safety was also evaluated in green color, as the reagents required are not known to cause serious injury and are not suspected carcinogens. Analytical Eco-Scale Assessment penalizes this method by 13 penalty points for reagents (1 penalty point (pp)) energy consumption (3 pp), operator safety (3 pp) and waste (6 pp) and ultimately recognizes the proposed method as an “excellent green analysis”.

#### Miniaturization Trends

A review of recently published analytical methods for the determination of boscalid and pyraclostrobin in strawberries indicates that most procedures are based on conventional QuEChERS protocols and have not been miniaturized. Nearly all the reviewed methods use standardized amounts of sample and solvents. Some methods [44,54,55] use higher amounts of sample or solvents. The standardized amounts were 10 g of sample and 10 mL of solvent (original and CEN QuEChERS) or 15 g of sample and 15 mL of solvent (AOAC QuEChERS). In comparison, Chu et al. [44] used 25 g of sample and 50 mL of extraction solvent. Mao et al. [54] used less sample (5 g); however, they used 20 mL of solvent. Ludvickova et al. [55] used only 1 g of sample; however, the sample was freeze-dried and for 100 g of freeze-dried strawberries, 1 kg of fresh strawberries was used. Therefore, the method is not truly miniaturized. Among the studies reviewed, the proposed method is the only one that simultaneously reduces both the sample amount and extraction solvent volume while maintaining the analytical performance required for routine pesticide residue analysis. Furthermore, unlike the reviewed methods, the proposed procedure combines sample and solvent miniaturization with the use of n-amyl acetate as a greener extraction solvent, a waste-derived carbonaceous sorbent for clean-up and GC-MS with mid-column backflushing, thereby improving both the environmental and operational sustainability of the analytical workflow without compromising analytical performance.

### 3.13. Real-Sample Analysis

The proposed method was applied to the screening of pesticide contamination in commercially available strawberries. A total of seven samples were purchased from local stores and markets and subsequently analyzed using the developed method. Out of the seven analyzed samples, three samples were positive for pesticide residues. For these real samples, the observed retention times fulfilled the acceptance criterion (deviation < ±0.1 min), and the relative ion abundance ratios of the qualifier ions remained within the tolerance limits specified by SANTE/11312/2021 v2026, confirming reliable analyte identification. In one sample, boscalid was detected at a concentration of 37.72 μg/kg, which exceeded the LOQ (Table 8). For the remaining two samples, the detected concentration of the pesticides was below the LOQ for boscalid. In all cases, the level of pyraclostrobin was below LOD. Pesticides were not detected in four samples. Importantly, none of the analyzed samples exceeded the maximum residue limits (MRLs) established for strawberries, indicating compliance with current regulatory requirements.

## 4. Conclusions

A novel analytical method for the determination of boscalid and pyraclostrobin in strawberries was developed and successfully validated. The results represent new outputs in the agrochemical, food safety and food monitoring areas and that of analytical chemistry. The proposed approach emphasizes analytical greenness using less-toxic reagents, reduced solvent and sample consumption and miniaturization of the QuEChERS procedure, resulting in lower organic waste generation. In addition, the method enables higher sample throughput, while maintaining robustness, safety and analytical efficiency, supporting sustainable laboratory practice. Method validation demonstrated good linearity, satisfactory accuracy and precision, and reliable performance in accordance with current regulatory guidelines. The method was successfully applied to the analysis of strawberry samples obtained from various sources in Slovakia, enabling effective screening of pesticide residues without exceeding established maximum residue limits. Overall, the developed method represents a sustainable and practical tool for routine pesticide residue analysis in strawberries and has the potential for broader application in food control laboratories.

## Figures and Tables

**Table 1:** Extraction recoveries (ER) and relative standard deviation (RSD) of pesticides using the original, CEN and AOAC versions of the QuEChERS.

Pesticide	Original (ER) * (%)	CEN (ER) * (%)	AOAC (ER) * (%)
Boscalid	96 (0.52) *	90 (2.8)	91 (1.1)
Pyraclostrobin	93 (28)	99 (13)	88 (25)

* intra-day precision values in % are given in brackets (*n* = 3).

**Table 2:** Greenness of explored solvents according to the Analytical EcoScale.

Solvent	GHS Symbols	Label	Penalty Points
Acetone	Flammable, Irritant	Danger	4
Acetonitrile	Flammable, Irritant	Danger	4
Amyl Acetate	Flammable	Warning	1
Ethyl Acetate	Flammable, Irritant	Danger	4
Hexane	Flammable, Irritant, Health hazard, Environmental hazard	Danger	8
Hexyl Acetate	Flammable, Environmental hazard	Warning	2

**Table 3:** Volume of obtained extract after shaking for 30 s and 60 s.

Vortex Time	30 s		60 s	
Mass of extract (g)	4.33	2.98	3.84	5.93
Volume of extract (mL)	4.94	3.40	4.38	6.77

**Table 4:** Volume of obtained extract after shaking for 15 s.

Experiment	1	2	3	4
Mass of extract (g)	7.32	7.32	7.22	6.83
Volume of extract (mL)	8.36	8.36	8.24	7.80

**Table 5:** Accuracy and precision overview of the method.

	Recovery 25 μg/kg	RSDr %	Recovery 125 μg/kg	RSDr %	Recovery 250 μg/kg	RSDr %	RSDwR % 25 μg/kg	RSDwR % 125 μg/kg	RSDwR % 250 μg/kg
Boscalid	83	13	88	6	92	9	9	6	4
Pyraclostrobin	75	6	81	4	91	11	10	12	8

**Table 6:** LOD, LOQ and coefficient of determination of the method.

	LOD (μg/kg)	LOQ (μg/kg)	R^2^
Boscalid	0.58	1.75	0.999
Pyraclostrobin	4.60	13.8	0.996

**Table 7:** Comparison of methods used for the analysis of boscalid and pyraclostrobin in strawberries.

Analytes	Sample Amount (g)	Extractant	Extraction	Clean-Up	Analysis	Recovery (%)	LOD (mg/kg)	AGREE Score	Ref
98 pesticides, incl. boscalid and pyraclostrobin	25 g	50 mL ACN	Chinese standard method		GC-MS/MS, HPLC-MS/MS	67.9–133.9	0.002–0.008 (instrument LOD)	0.37	[44]
16 pesticides, incl. boscalid and pyraclostrobin	10 g	10 mL ACN	Modified MWCNT QuEChERS	5 mg MWCNT sorbent	GC-MS/MS LC-MS/MS	100.5–117.2 (B) 94.9–111.6 (P)	0.0003 (B) 0.0002 (P)	0.42	[45]
30 pesticides incl. pyraclostrobin	10 g	10 mL ACN	CEN 15662 QuEChERS		LC-LTQ/Orbitrap MS	82.1–97.7	0.0005 (MDL)	0.45	[46]
Pyrimethanil, boscalid	10 g	10 mL ACN	Optimized QuEChERS [47]	25 mg/mL PSA and 25 mg/mL C18, 0.22 μm filter	GC-MS/MS	81.2–89.4	0.00167–0.030	0.4	[48]
182 pesticides, incl. boscalid	Not explicitly stated	ACN	QuEChERS	No additional clean-up, EtOAc FS extract added as analyte protectant	GC-MS/MS	70–121 (B)	0.0033	0.35	[49]
4 pesticides, incl. pyraclostrobin	10 g	10 mL	Optimized QuEChERS [47]	No additional clean-up	GC-MS/MS, LC-MS/MS	83.8–98.3	0.0008	0.42	[50]
36 pesticides incl. boscalid and pyraclostrobin	10 g	10 mL ACN	Citrate QuEChERS	1.5 mL of dSPE extract + 50 μL 5% FA in ACN and evaporated, reconstituted in toluene	GC-MS/MS	0.020 ± 0.003 (B) 0.040 ± 0.005 (P)	n/a	0.44	[51]
Boscalid, pyraclostrobin	10 g	10 mL	LLE with Acetonitrile	dSPE with PSA	Any	-	-	0.43	[34]
430 residues including boscalid and pyraclostrobin	10 g	10 mL ACN	EN-15662	dSPE with PSA	LC-MS/MS and GC-MS/MS	Between 70 and 120%	<0.005	0.45	[52]
275 residues including boscalid and pyraclostrobin	15 g	15 mL	AOAC QuEChERS	dSPE with PSA	LC-MS/MS	79.4% (B, 0.01 mg/kg) 88.1% (P, 0.01 mg/kg)	0.007 (B) 0.005 (P)	0.45	[53]
26 pesticides including boscalid and pyraclostrobin	5 g	20 mL	Modified CEN QuEChERS	dSPE with PSA, C18 and GCB	LC-MS/MS	79% (B, 0.01 mg/kg) 98% (P, 0.01 mg/kg)	Not explicitly stated	0.44	[54]
484 pesticides	1 g freeze dried strawberries + 10 mL water (1% formic acid)	10 mL ACN	QuEChERS	dSPE with PSA (only for GC-MS/MS)	GC-MS/MS and LC-MS/MS	Not explicitly stated	Lowest successfully validated concentration level	0.42	[55]
Boscalid, pyraclostrobin	6 g	3 mL Amyl acetate	mini-QuEChERS	dSPE with PSA, C18 and carbonaceous sorbents [32]	GC-MS	92% (Boscalid) 91% (Pyraclostrobin)	0.00058 (B) 0.00460 (P)	0.56	This work

**Table 8:** Real-sample analysis results.

Sample No.	Findings
1	Boscalid 37.72 μg/kg
2	<LOQ
3	<LOQ
4	<LOD
5	<LOD
6	<LOD
7	<LOD

Note: LOQ—limit of quantification, No.—number of the sample.

## Data Availability

The original contributions presented in the study are included in the article, further inquiries can be directed to the corresponding author.

## Abbreviations

The following abbreviations are used in this manuscript:

ACN	Acetonitrile
AESA	Analytical Eco-Scale Assessment
AGREE	Analytical Greenness Metric Approach
ANOVA	Analysis of Variance
AOAC	Association of Official Analytical Communities
C18	Silica Gel modified by Octadecyl
CEN	European Committee for Standardization
Complex MoGAPI	Complex Modified Green Analytical Procedure Index
DAD	Diode Array Detector
dSPE	dispersive Solid Phase Extraction
ER	Extraction Recovery
FA	Formic Acid
GC	Gas Chromatography
GC-MS	Gas Chromatography Mass Spectrometry
GHS	Globally Harmonized System of Classification and Labeling of Chemicals
HPLC	High-performance Liquid Chromatography
LLE	Liquid–Liquid Extraction
LOD	Limit of Detection
LOQ	Limit of Quantification
MDL	Method Detection Limit
ME	Matrix Effect
MF	Matrix Factor
MMI	Multimode Inlet
MRL	Maximal Residual Limit
MS	Mass Spectrometry
MS/MS	Tandem Mass Spectrometry
MSD	Mass Selective Detector
MWCNT	Multi-Walled Carbon Nanotubes
PSA	Primary-Secondary Amine
QuEChERS	Quick, Easy, Cheap, Efficient, Rugged, Safe
RSD	Relative Standard Deviation
SIM	Selected Ion Monitoring

## Appendix A

**Table A1:** ANOVA of extraction recoveries (ERs) for boscalid using various solvents.

Groups	Count	Sum	Average	Variance
Ethyl Acetate	4	418.8217	104.71	20.28
Acetone:Hexane	4	225.4261	56.36	4234.89
Acetone	4	361.5325	90.38	233.18
Hexane	4	176.877	44.22	4.00
Amyl Acetate	4	335.6994	83.92	80.95
Hexyl Acetate	4	1302.889	325.72	4051.54

**Table A2:** ANOVA of extraction recoveries (ERs) for pyraclostrobin using tested solvents.

Groups	Count	Sum	Average	Variance
Ethyl Acetate	4	593.0968	148.27	1553.61
Acetone:Hexane	4	270.1308	67.53	6093.50
Acetone	4	367.9736	91.99	672.134
Hexane	4	481.0684	120.27	7296.94
Amyl Acetate	4	307.1539	76.79	447.873
Hexyl Acetate	4	992.7871	248.20	27,724.8
